# Design and Calibration of an Organic Diffusive Probe to Extend the Diffusion Gradient Technique to Organic Pollutants

**DOI:** 10.3390/ijerph8083318

**Published:** 2011-08-15

**Authors:** Antonina Bondarenko, Daniela Sani, Maria Letizia Ruello

**Affiliations:** 1 Department of Chemical Technology, Lipetsk State Technical University, 398600 Lipetsk, Russia; E-Mail: antonina_vlad@lipetsk.ru; 2 Dipartimento di Fisica e Ingegneria dei Materiali e del Territorio, Università Politecnica delle Marche, 60131 Ancona, Italy; E-Mail: d.sani@univpm.it

**Keywords:** pollutant mobility, DGT, persistent organic pollutant, passive sampling, risk assessment in soil

## Abstract

The objective of this study was to develop a method for measuring the mobility of persistent organic pollutants in the solid phase of soils within the context of environmental pollution risk assessment. A new diffusive probe, purposely designed by adapting the diffusive gradient technique method, measures labile organic species by immobilizing them after diffusion through a thin deionized water layer. The measure of the mass accumulated is used to calculate the flow of pollutant from solid phase to pore water. Naphthalene was chosen as a model persistent organic pollutant. The probe was calibrated at different temperatures and was then tested in several microcosms at different porosity and reactivities with naphthalene (one clay soil, two sandy soils and one natural soil). The probe response showed good agreement with the expected different abilities of the solid phases in restoring the solution phase. The concentration of naphthalene in the pore water was well buffered by rapid equilibria with the solid phase in the investigated natural soil. In contrast, pore water concentration in the sandy soils decreased rapidly and the flow was slackened, especially for the sandy soil with finer particles. In clay, only a fraction of the total naphthalene content was present in the labile fraction, while the remaining was tightly bound and was not released to the pore water. Therefore, this first stage of testing points out that the diffusive gradient technique, if optimized, can properly quantify the mobility of organic pollutants in soil.

## 1. Introduction

Existing rules and guidelines on pollution risk assessment are primarily based on total concentrations in soil and in ground water [1–4]. These lump sum measurements, although undoubtedly useful, are quite far from providing any information on pollutant lability and the risk associated with their mobility in soils and ground water. Moreover, researchers have recognized that the total content of persistent pollutants (both organic and heavy metal) includes large fractions that are unavailable to plants, microorganisms, or soil fauna [5]. In fact, soil properties can modify pollutant bioavailability by conditioning the equilibrium between solid and liquid phases, hence altering the amount of exposure to a molecule and reducing the impact of the total loading [6]. Because many processes affect the equilibrium between solid and liquid phases, including diffusive and convective transport and the influence of root microenvironments and exudates [7], it is unlikely that any single chemical surrogate could mimic bioavailability, unless one process is dominant.

Batch techniques give results dependent on solvent/soil ratios, shaking time, and solvent strength. Extraction techniques provide a simple classification of the fractions of soil pollutants [8], but these are based on arbitrary responses to chemical reagents rather than on a true reflection of pollutant lability. Therefore, both these techniques cannot provide detailed information on the transportation of molecules from soil with the solution, which is important for assessing bioavailability and the risks associated to pollutant mobility [9].

Measurements of pollutant concentration in pore water seem to provide a better indication of bioavailability [10]. In fact, extraction of pore water from soil isolates the aqueous phase to which plant roots and microorganisms are exposed. However, these measurements fail to take into account the ability of the soil to sustain the solution concentration following depletion by uptake or biodegradation.

Any procedure that separates solution and solid phase inevitably disrupts the physical-chemical equilibrium, which may affect the distribution of species in solution [11]. Ideally, the best way to measure pollutant concentration in pore water is by means of *in situ* procedures which either minimize disturbance or perturb the solution in a controlled way.

A passive sampling technique—the diffusion gradients in thin-films (DGT) technique—able to measure heavy metal labile flux from solid to solution phase, has been developed and extensively investigated in the last two decades [12–17]. This technique acts as a chemical-physical sink for pollutants, lowering local pollutant concentrations and mimicking biota uptake. It allows the precise calculation of both fluxes and pore water concentration taking advantage of the diffusion process. This is the basis of the technique of diffusive gradient in thin-films (DGT) which is founded on kinetic rather than equilibrium principles. In the last two decades, passive sampling methods have shown much promise as tools for measuring aqueous concentrations of a wide range of priority pollutants [18–20]. Passive dosimeters have been successfully used to monitor organic and inorganic contaminants in air, water, sediment, and soil. However, none of the passive probes for organic contaminants in soil have been used under the dynamic conditions on which the DGT technique is based.

In this paper, the authors present a new organic diffusive probe (ODP) able to measure organic pollutant concentration in soils by perturbing the equilibrium between solid and liquid phases in a controlled way. The new probe, ODP, is designed by adapting DGT, originally meant for heavy metals, to organic molecules. The main modifications relate to:

1. the material suitable as organic pollutant receptor;
2. the shape of the overall testing device;
3. the materials filling the probe.

In particular, the used receptor materials are: activated carbon or cyclohexane. The ODP was calibrated and tested on several model soils (*i.e.*, microcosms), namely: one clay soil, two sandy soils and one natural soil. Naphthalene was chosen as pollutant tracer: it is the smallest of polycyclic aromatic hydrocarbon, a class of persistent organic pollutants that is often present in industrial contaminated sites. No modification was introduced in the applied diffusion theory and in the calculation of concentration.

The experimental section is organized in three parts. The first two are dedicated to the new method itself by describing the new device—ODP assembly and calibration—and the adopted analytical procedures. The third one is mostly dedicated to testing the method under controlled conditions, *i.e.*, in controlled microcosms and pollutants.

## 2. Experimental Section

### 2.1. Probe Assembly and Calibration

**Figure 1.**
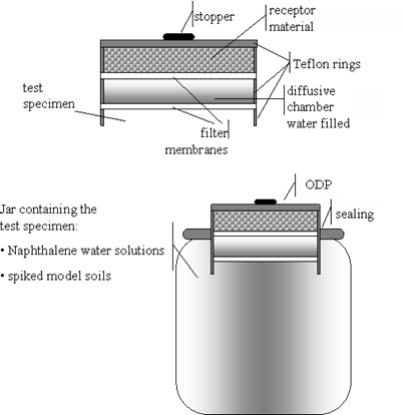
Organic diffusive probe (ODP) and testing device: schematic representations (not to scale).

The main features of the new organic diffusive probe, modified for sampling organic pollutants in a soil matrix, can be broken down as follows (Figure 1):

- The device, of low organic reactivity, is made up of three modular polytetrafluoroethylene (Teflon) rings (external diameter = 37.0 mm and internal diameter = 32.2 mm); the first ring is in contact with the specimen to be tested, the second—filled with deionized water—acts as a diffusive chamber, the third hosts the receptor material;
- The layer of material is suitable as an organic pollutant container—two receptor materials were evaluated: activated carbon or cyclohexane;
- The diffusive chamber—water filled—(volume = 8.55 cm^3^; surface area, *A* = 8.14 cm^2^; thickness, *Δg* = 1.05 cm), were bordered by two filter membranes (glass microfibre GF/A, pore 1.6 μm, diameter = 37 mm).

To perform the tests, the ODP was assembled and then inserted at the top of the testing apparatus, as illustrated in Figure 1. The apparatus consisted of: 0.5 L glass jar; open-top Teflon cap; O-ring seal; organic diffusive probe (ODP).

The principle of measurement for the probe is based on Fick’s first law as the traditional diffusion-adsorption technique [15]. The receptor material was separated from the specimen by a water chamber of thickness *Δg* (Figure 1). Organic molecules diffuse through the water layer and are rapidly bound by the receptor material, so their concentration at the interface water-receptor is maintained at zero throughout the deployment. Water chamber and glass fiber membranes were chosen specifically in order not to interpose selective media for the pollutant diffusion process [19]. The ODP measures the average flux of pollutant that diffuses through the water layer by accumulating a mass of pollutant over time through a well-defined area.

**Figure 2.**
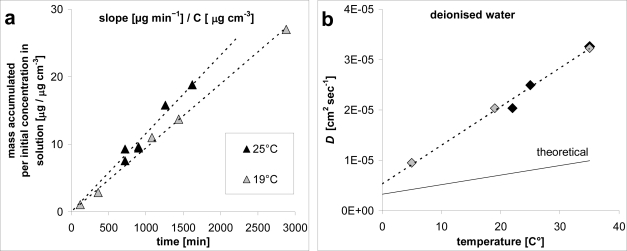
Calibration of the ODP by calculating the diffusion coefficient (*D*). (**a**) Linear regression of the experimental data to obtain the slope (the data for only two temperatures are shown as an example). (**b**) Relations of *D* with temperature; each value of *D* is calculated with Equation (1); black symbols indicate tests with activated carbon, grey symbols indicate tests with cyclohexane as receptor material.

The probe was calibrated by calculating the diffusion coefficients (*D*) of naphthalene as perceived by the ODP itself. *D* was measured by using a simple diffusion test. For the calibration test, the jar was overfilled with a water solution of naphthalene (Figure 1) at known concentration (average value 23 μg cm^−3^). The diffusion coefficient, *D* (cm^2^ s^−1^), was calculated using Equation (1) [21,22]:

(1) D=slopeΔg/AC

where *A* (8.14 cm^2^) is the exposed area of diffusion chamber of thickness *Δg* (1.05 cm); *C* (μg cm^−3^) is the concentration of the naphthalene solution (typically about 23 μg cm^−3^); *slope* (μg sec^−1^) was obtained by plotting the amount of naphthalene accumulated versus time. In particular, to determine *D* for each temperature, at least three determinations were performed, ranging between 120–2880 minutes (Table 1, Figure 2a).

**Figure 3.**
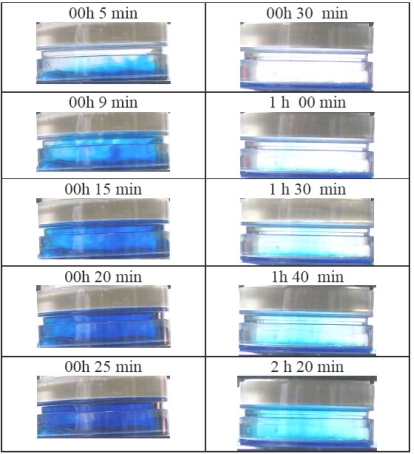
Methylene blue diffusion under stirred (left side) and unstirred (right side) conditions. The images focus the methylene blue diffusion in the diffusive chamber.

The diffusion test may be conducted by keeping the solution in the jar either stirred or unstirred. After analyzing the results suggested by an appositely designed test, the unstirred condition was preferred. This test consists of exposing the ODP to a solution of methylene blue in water (0.005% mass) in stirred and unstirred conditions. The phenomenon of methylene blue transport through the water layer was registered photographically (a transparent probe in polycarbonate was used). The results are shown in Figure 3. Turbulences in the diffusive chamber due to the stirring procedure raised the solute transport (Figure 3 left side) and the optical density (visually estimated); they became equal to bulk solution after 25 minutes. In contrast, the unstirred condition colored the diffusive chamber in 180 minutes and the optical density was still lower than bulk. Therefore, according to the series of images, the unstirred condition seemed to better reproduce a “pure diffusive” environment in the diffusive chamber and was therefore adopted in our method.

The diffusion coefficient, calculated with Equation (1), also depends on the temperature at which the experiment is performed. Consequently, the influence of the temperature has been studied for ODP calibration. The influence was investigated by taking measurements in a thermostatic box ranging between 5–35 °C (±2).

In Figure 2b, the obtained results are compared with the theoretical ones [23]. The experiments conducted with activated carbon (black symbols in Figure 2b) and with cyclohexane (grey symbols in Figure 2b) as receptor (see also Section 2.2 below) provided very similar values of *D*. However, the diffusion of naphthalene inside the water chamber showed stronger dependence on temperature if compared with the theoretical values [23]. In particular, the observed *D* values were greater and increased faster than the corresponding values described in literature (Figure 2b). The causes of this unexpected result were not investigated further, mainly because it was considered beyond the scope of our work, its focus being an investigation of ODP.

### 2.2. Receptor Materials and Analytical Methods

In general, some basic properties are expected of a material for it to be used as a receptor: high adsorptive activity at a small concentration of target pollutant, high diffusivity (*i.e.*, homogeneous distribution of the concentration inside), stability in water and easy handling for its use in the probe. Moreover, the receptor material should show an absence of irreversible adsorption and of analytical interference during analytical checks. The performances of several adsorptive materials were investigated: activated carbon, polymeric material, organic solvent, natural alumino silicates and silanised silica gel. They mostly show that each of the investigated materials can potentially be used as a receptor layer. However, activated carbon (Anasorb CSC) and organic solvent (cyclohexane) were adopted because they are free from interference and easier to manipulate than other materials.

In particular, Anasorb CSC (surface area 1100 m^2^ g^−1^, total volume of pores 0.25–0.40 cm^3^ g^−1^) was chosen for its adsorptive properties, stability in water, good analytical control and maneuverability. The amount of carbon in the probe was 2 g. The carbon was water saturated before its use in the probe. Cyclohexane (Carlo Erba, HPLC grade) was adopted for its solvent properties towards naphthalene, immiscibility in water, possibility of direct gas chromatographic (GC) analysis. The amount of cyclohexane in the probe was 6.6 g.

Different analytical methods were adopted to determine the amount of naphthalene when spiked in water solution (*i.e.*, in the jar) and when accumulated in the receptor layer. The naphthalene concentration in water solution was determined by fluorimetry (Perkin Elmer LS50) using the following setting: wavelength Em λ 330 nm, Ex scan range 250–320 nm, slits Em and Ex 5 mm, Ex signal registered at 285 nm.

The naphthalene mass accumulated in the receptor material was determined by GC analysis (GC 8000 top CE Instruments): analytical column HP-5 (crosslinked 5% HP ME siloxane, 25 m × 0.32 mm × 0.52 μm film thickness); temperature range 80–220 °C, heating step 15 °C/min; detector FID; split injection, internal standard hexadecane 8.9 μg cm^−3^.

In the case of carbon, it was separated from the excess water after the test, mixed with CS_2_ (5 mL of CS_2_ per 1 g of activated carbon) and sonicated at room temperature for 30 minutes before GC analysis. The reproducibility of this procedure was tested on eight samples in the range 2.9–23.2 μg cm^−3^. The obtained results showed good reproducibility (standard deviation 1.1), although the extraction efficiency was low (average 38%). This efficiency of extraction is close to the values that DiGiano obtained for aromatic hydrocarbons recovered by carbon disulfide extraction after passive sampling in water with activated carbon [18]. The same author obtained an efficiency of extraction equal to 75% for the recovery of atrazine from activated carbon by methylene chloride extraction. It is clear that the detection of low analyte concentrations and other pollutants, requires specific task. For this reason the problems linked to optimization of the probe was not investigated in this first tentative approach to organic molecules with DGT technique.

In the case of cyclohexane as a receptor it was directly analyzed by GC. The possibility of using a receptor that can be directly analyzed is very interesting for two main reasons: firstly, because direct analysis avoids any artifact due to sample preparation, and secondly, because it is possible to analyze it without disassembling the ODP. At fixed time intervals, 2 μL of cyclohexane was sampled by GC syringe from the upper face of the probe (from the hole of the stopper, see Figure 1) and analyzed (internal standard was directly added to cyclohexane before adding it to the third ring of the ODP). The possibility of a multiple analysis is particularly interesting in order to observe the trend of the pollutant resupply in time and to obtain kinetic information on solid solution equilibria.

The results obtained from the test of calibration of the probe demonstrated the equivalence of using activated carbon or cyclohexane as a receptor. For the reasons above described, cyclohexane was preferred and then the subsequent experimental work, reported here, used only this chemical.

### 2.3. Tests on Controlled Microcosms

Several types of model soils were used to test the performance of the ODP. In particular, two sandy soils, one pure clay and one natural soil were selected to make controlled microcosms (*i.e.*, model soils spiked with naphthalene). The sandy soils were washed with tap water and air dried in order to remove the finest particles. The pure clay soil, namely a bentonite, was used as received. The natural soil was air dried and sieved at 2 mm. It has been classified as silty clay [24], non-contaminated top soil sampled at a depth of 0.5 m.

Model soils were characterized by water vapor isotherms, the capacity of their adsorptive monolayer, their surface area and their limited sorption volume. The model soils were spiked with naphthalene to prepare microcosm samples. The methods for preparing microcosm samples were different depending on the different adsorptive capacity of model soils: the sandy soils were saturated in naphthalene and kept in water solution for 24 h at 25 μg cm^−3^; whereas the bentonite and natural soil were saturated in two steps: pre-saturation in a desiccator with naphthalene vapor and then saturation in a water solution (25 μg cm^−3^ 24 h).

Tests on microcosm samples were performed with the apparatus described in Section 2.1 (Figure 1) with the jar containing the microcosm sample. To avoid unknown effects associated with unsaturated soils, measurements were made on a microcosm sample, the moisture content of which was increased up to 100%.

To avoid air bubbles and to ensure that the overall probe surface was exposed, a small amount of microcosm sample was gently smeared on the first filter membrane of the probe (Figure 1). Then, the ODP was inserted in the open-top cap (Figure 1). Tests were performed in an air conditioned room at 20 ± 4 °C.

To quantify naphthalene diffusion in microcosm samples the naphthalene mass (μg) accumulated in the receptor material (cyclohexane) was periodically measured between 3 to 350 hours. As the accumulated mass depends on the initial concentration of naphthalene in microcosm pore water, the concept of *apparent volume* was introduced. It is defined as the ratio of the accumulated mass of naphthalene (μg) per the initial concentration of naphthalene in microcosm pore water (μg cm^−3^). This is not the actual volume of pore water extracted, but it is the volume of pore water (at a given concentration) that should be “virtually” sampled to produce the accumulated mass. We named this volume “*apparent*” because the mass of naphthalene accumulated in the sampler reflects not the sampling of a certain volume of pore water at a given concentration, but reflects the replenishment of soil water concentrations by diffusion and desorption from bound sites when the pore water concentration is reduced as the sampler accumulates naphthalene. The amount of desorption from these bound sites will vary depending on the nature of the binding sites. The *apparent volume* provides values independent of analyte concentration in water. The initial analyte concentration was the same inside the data set of each soil material, but was different between different soil. The use of *apparent volume* allowed to compare results from different microcosms in terms of the effective replenishment of soil water concentrations.

## 3. Results and Discussion

**Figure 4.**
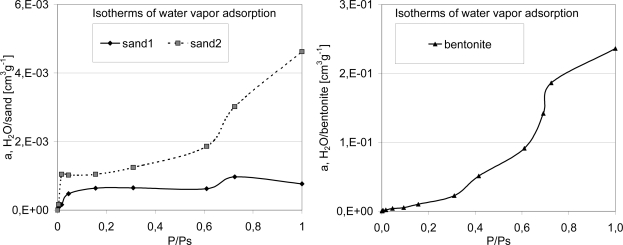
Isotherm of water vapor adsorption of the material used for testing the ODP performance.

To know the properties of the model soils is useful in predicting the behavior of naphthalene and evaluating the performance of the ODP on the basis of the expected responses. In particular, the water vapor isotherms (Figure 4), the capacity of adsorptive monolayer, surface area and limited sorption volume (Table 2) are useful to determine and compare their adsorptive properties.

**Figure 5.**
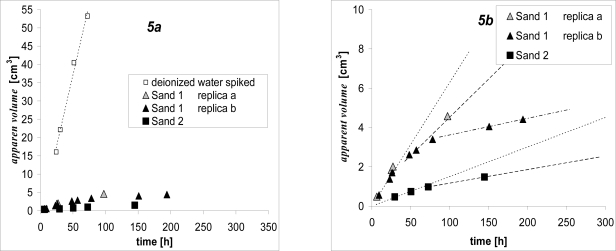
Trend in time of the apparent volume sampled by the ODP in microcosms with *Unsustained* flux. (**a**) All the samples are compared with the apparent volumes measured in free solution of naphthalene (deionized water spiked). (**b**) The dotted, dashed and dotted-dashed lines are regression lines on small range of time, these lines point out the apparent volume continuing to increase more and more slowly over time. For Sand 1 there are only three lines because “replica a” and “replica b” were interpolated as one data set.

**Figure 6.**
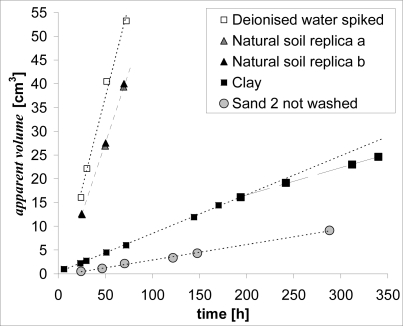
Trend in time of the apparent volume sampled by the ODP in microcosm samples with *Partially Sustained* and *Fully Sustained* flux. Two lines are drawn for clay (the steeper line dotted, and the shallower line solid) to point out a lowering of flux due to presence of a fraction of naphthalene adsorbed in a kinetically inert manner.

The overall result set, based on the analysis of cyclohexane samplers, is presented in Figures 5 and 6. The resulting discussion refers to the apparent volume measured in the free solution of naphthalene at 20 °C, as the diffusion process in this system (only deionized water spiked with naphthalene) is unaffected by soil interactions.

Each investigated microcosm sample showed its own specific trend of the naphthalene flux, that depended on soil interactions with pore water. In fact, the trend of the apparent volume in time is linear for spiked deionized water, but shows different shapes for microcosms (Figures 5 and 6).

Test results on Sand 1 and 2 are presented in Figure 5. They are also compared with those on deionized water in Figure 5a. The following main points can be outlined from the results:

- Qualitatively, the overall process seems very slow compared with deionized water. The apparent volume did not increase linearly over time for Sand 1 or for Sand 2, even though no significant adsorptive capacity can be ascribed to either sandy soil.
- Quantitatively, Sand 1, having particle size five times greater than in Sand 2 (Table 2), after 50 hours, was able to transport about 3 cm^3^ of the apparent volume, while Sand 2 transported less than 1 cm^3^.

Generally, in the absence of evident adsorption capacity, only the diffusion process can be responsible for naphthalene transport from the bulk soil to the probe, depending on the porosity and the tortuosity of the microcosm matrix. Both parameters depend on particle size, dimension and shape and, obviously, Sand 1 has a better capacity to diffuse naphthalene. Moreover, the non linearity of the naphthalene flux could either be ascribed to a microcosm matrix or associated to the capacity of the microcosm to resupply the naphthalene. In the presence of an inert microcosm like sand, only diffusion can restore the depleted concentration near the probe. This effect, most evident in Sand 1, but present in both sands, causes the apparent volume growth to increase more and more slowly over time (Figure 5b). The behavior, observed for persistent organic pollutants (like naphthalene), is consistent with the so called *Unsustained* flux, broadly referenced in the main literature for trace metal [15]. In all these cases there is no resupply from the soil matrix to the bulk solution. The supply of pollutant to the diffusive device is solely by diffusion from the pore water, which progressively depletes, first near the probe, and later in the overall specimen. The flux consequently declines with the deployment time.

If the microcosm has adsorptive capacity, then the process of resupply is controlled by diffusion and desorption as well. The transport of naphthalene to the probe is the combination of both the mechanisms in a complex equilibrium of the two kinetics.

The desorption effect produced by the presence of fine particles in Sand 2 (in a modest percentage) when used unwashed, is the reason for the higher values of the apparent volume (Figure 6). In this case, the observed behavior is consistent with the so called *Partially Sustained* flux, broadly referred to in the main literature regarding trace metals [15]. The desorption of solutes from soil to solution is present, but the resupply is partial because it is insufficient to sustain the initial bulk concentration when depleted by the probe.

Similar results were obtained from clay (Figure 6), but in a more complex equilibrium between adsorption/desorption because of its nature: bentonite has high adsorption capacity but also great adsorption strength.

The clay matrix adsorbed naphthalene during the saturation step and then desorbed the most labile fraction when, during deployment, pore water concentration was lowered by the probe. Although the clay matrix was very close (particle size <0.075 mm; *i.e.*, low porosity and high tortuosity), the naphthalene flux was higher than for sands and also linear for a long time. At 50 hours the apparent volume was about 5 cm^3^ and the trend stayed linear for until 200 hours (about 8 days). The long time requested for the desorption is an indirect measure of how tightly naphthalene is attached to clay particles. After this period, the labile pool decreased causing a lowering of flux because the clay adsorbed a fraction of naphthalene in a kinetically inert manner. This behavior of contaminants in soil is well known [25] and it is interesting to have the tools to measure and monitor it. Contaminant fractions, upon interaction with soil matter, become progressively less available due to transport into soil aggregates and active microsites (binding of contaminant to mineral and organic matter). It would be interesting to incorporate the results derived from these kind of investigations into risk assessment and remediation protocols when the potential of contaminants to be released from soil matrices are evaluated. However, new protocols should be approached with caution, because this could be a reversible process in which contaminants are released over time due to a change in sorbent properties, the slow migration of compounds to accessible sites or when the contaminant changes into an available form.

Surprisingly, the highest flux of naphthalene was recorded in the natural soil. The trend in time of the apparent volume was very close to that measured in free solution of naphthalene in deionized water (Figure 6). This condition reproduced the so called *Fully Sustained* flux [15]: pollutants removed from the pore water by the probe were rapidly resupplied by the soil matrix and the concentration in solution was effectively buffered to a constant value.

The reason why natural soil showed a fully sustained flux compared to the partially sustained flux showed by clay was not thoroughly investigated, but some general considerations may be pointed out. Firstly, the porosity and the tortuosity of the clay matrix offer a higher resistance to the naphthalene flux than those of the natural soil. Secondly, the complex equilibrium between sorption/desorption and depletion/resupply depends on a number of factors influencing the real diffusion coefficient of naphthalene which therefore drives the real flux trough the soil matrix. Thirdly, it is well known that bentonite clay has a strong adsorption capacity and strength. Clay’s capacity to retain adsorbed naphthalene should be assumed to be much higher than the labile form offered by natural soil.

In any case, conditions like that of naphthalene adsorbed in labile form by the investigated natural soil must be considered more dangerous for the environment as the pollutant is fully available for migration and transport in the ground.

## 4. Conclusions

The present paper aimed to describe the activities performed to test a method for measuring the mobility of persistent organic pollutants in the solid phase of soils within the context of environmental pollution risk assessment. The method is based on the design of a new probe for the passive sampling of organic pollutant in soils. The probe, namely an organic diffusive probe (ODP), was prepared to reproduce diffusion gradients in thin-films (DGT). This method could be especially useful due to its cheap and simple investigation conditions. It should be considered as a first attempt in expanding DGT, largely employed in water, sediment and soil with metals, to other field of investigations like the organics pollutants.

In terms of naphthalene flux, the results reproduced the so called *Unsustained*, *Partially Sustained* and *Fully Sustained* typical conditions of the trace metal measurements in DGT very well. The successful determination of naphthalene resupply from solid to liquid phase in soil illustrates the wide scope of ODP and the potential for its adoption for a wide range of applications, including organic persistent contaminant.

In this work, a water-filled diffusive chamber was adopted to avoid any interference from the diffusion material (membrane or gel) and to focus on the response of the microcosm to pollutant depletion. Other materials can be exploited inside ODP to make the diffusive layer selective for particular target pollutants.

The results also demonstrated the equivalence of using a solid or liquid material as a receptor. Although in this work the use of a liquid receptor allowed convenient working conditions in the laboratory, a wide range of different adsorbent materials (for example chromatographic phase) can be successfully introduced in the ODP. More specific adsorbent materials could improve the performances of the ODP and confidence on field conditions.

At any rate, the nature of the materials used in the ODP or its configuration should not be considered to be the real goals of further research, especially if compared with the difficulties and challenges offered by the *in situ* investigation on a soil matrix. This opportunity was not exploited in the present paper, because the direct *in situ* sampling is still seldom adopted, even in the classic DGT technique for heavy metals [26]. Both the DGT and ODP probes require sampling with a water content near to saturation [27]. Such conditions are rarely presented in the field soil environment, especially not for long periods. However, the probe can be used in conditions close to field conditions, for instance, in a laboratory, directly on the undisturbed core of soil hydrated at or above 80% field capacity.

To fully exploit the potential of this technique it is also necessary to develop a robust numerical model for organic substances to interpret the kinetic information provided by ODP with a view to risk assessment procedure. We suggest that the goal of this technique will only be reached through interdisciplinary work.

## Figures and Tables

**Table 1.** Experimental values of diffusion coefficient (*D*).

**Temperature (°C)**	***D* (cm^2^ sec^−1^)**	**Regressive equation**	**n**	**R^2^**	**Material**
5	9.50E-06	y = 7.368E-05x	6	9.957E-01	Cyclohexane
19	2.03E-05	y = 1.577E-04x	5	9.977E-01	Cyclohexane
35	3.22E-05	y = 2.500E-04x	4	9.632E-01	Cyclohexane
22	2.03E-05	y = 1.577E-04x	5	9.977E-01	Carbon
25	2.49E-05	y = 1.932E-04x	6	9.548E-01	Carbon
35	3.26E-05	y = 2.525E-04x	3	9.984E-01	Carbon

x (sec) = sampling time; y (cm^3^) = amount of naphthalene (μg) accumulated at prefixed time divided for the concentration of naphthalene (μg cm^−3^) in the test solution.

**Table 2.** Properties of the materials used as model soils.

**(a)**				
**Material**	**Particle size (mm)**	**Adsorptive monolayer capacity (mmol g^−1^)**	**Surface area (m^2^ g^−1^)**	**Limited sorption volume (cm^3^ g^−1^)**
Sand 1	0.85–1.75	0.035	2.7	0.0009
Sand 2	0.21–0.355	0.056	4.2	0.0046
Clay (bentonite)	<0.075	1.39	105	0.253

**(b)**					
**Material**	**Particle size (mm)**	**Fine fraction (%)**	**Porosity (−)**	**pH**	**EC (μS/cm)**
Natural soil (silty clay)	<0.05	76	0.55	8.16	221
